# Prevalence of *Plasmodium falciparum* and *Salmonella typhi* Infection and Coinfection and Their Association With Fever in Northern Tanzania

**DOI:** 10.24248/EAHRJ-D-18-00006

**Published:** 2018-11-23

**Authors:** Jaffu Chilongola, Sophia Kombe, Pius Horumpende, Rebeka Nazareth, Elias Sabuni, Arnold Ndaro, Eliakimu Paul

**Affiliations:** a Department of Biochemistry and Molecular Biology, Kilimanjaro Christian Medical University College, Moshi, Tanzania; b Clinical Trials Department, Kilimanjaro Clinical Research Institute, Moshi, Tanzania; c Kilimanjaro Christian Medical Centre, Moshi, Tanzania

## Abstract

**Background:**

*Plasmodium falciparum* and *Salmonella typhi* are major causes of fever in the tropics. Although these infections are caused by different organisms and are transmitted via different mechanisms, they have similar epidemio-logic and clinical features. This study aimed to determine the prevalence of *S. typhi* and *P. falciparum* infections and their associations with fever at 2 sites in Northern Tanzania.

**Methods:**

This was a community-based, cross-sectional study, conducted from February to June 2016, involving 128 randomly selected individuals, aged between 1 and 70 years. Sixty-three (49.2%) participants were recruited from Bondo Ward, Tanga Region, and 65 (50.8%) were recruited from Magugu Ward, Manyara Region. Blood samples were collected by venepuncture into sterile microtubes. Detection of pathogen DNA was achieved via a multiplex real-time polymerase chain reaction assay. Data analysis was done using Stata, version 14. Prevalence data were presented as numbers and percentages, and chi-square analysis was used to assess associations. P values of .05 or less were considered statistically significant.

**Results:**

Of 128 participants, 31 (24.2%) and 17 (13.3%) tested positive for *P. falciparum* and *S. typhi* infection, respectively. Of the 63 participants from Bondo, 31 (49.2%) had *P. falciparum* parasitaemia. None of the participants from Magugu tested positive for *Plasmodium* parasitaemia. *S. typhi* bacteraemia was detected in 11 (17.5%) of 63 and 6 (9.2%) of 65 participants in Bondo and Magugu, respectively. *P. falciparum–S. typhi* coinfection was only detected in Bondo (n=6, 9.5%). Age was the only variable that showed a significant association with both *P. falciparum and S. typhi* infection; falling within the 5-to 9-year or 10-to 15-year age groups was associated with both infections (X^2^=2.1; *P*=.045). Among the 30 patients with *Plasmodium* parasitaemia, 7 (23.3%) had fever, whereas 2 (12.5%) of 16 patients infected by *S. typhi* had fever. *P. falciparum* infection (X^2^=12.4, *P*<.001) and *P. falciparum–S. typhi* coinfection (X^2^=5.5, *P*=.019) were significantly associated with fever, while *S. typhi* infection alone was not.

**Conclusion:**

*S. typhi* and *P. falciparum* were considerably prevalent in the area. One-third of the *P. falciparum–S. typhi* coinfected individuals in Bondo had fever. *P. falciparum* infection was an important contributor to febrile illness in Bondo. In the presence of coinfections with *P. falciparum* and *S. typhi*, the use of malaria rapid diagnostic tests should be emphasised to reduce irrational use of medications.

## INTRODUCTION

In the investigation of fever in the tropics, 2 important diagnoses to be ruled out are typhoid fever and malaria. Both cause significant morbidity, mortality, and economic loss. Despite the progress in controlling salmonellosis globally, typhoid fever remains a public health problem in several parts of the world. Despite a reported decline in incidence in many countries, the disease is still widespread, particularly in South Asia and sub-Saharan Africa, as a source of significant morbidity, mortality, and economic losses.^1,2^ The World Health Organization (WHO) estimates the global typhoid fever disease burden at 11 to 20 million cases annually, resulting in about 128,000 to 161,000 deaths per year.^3^ Malaria affects about 1 billion people each year, out of which 1 to 3 million die. In the tropics, *S. typhi* and *Plasmodium falciparum* are the most endemic pathogens,^1,4^ causing typhoid fever and malaria, respectively. The 2 diseases are associated with poverty and underdevelopment, making them more prevalent in underdeveloped countries.^5,6^ Although these diseases are caused by vastly different organisms transmitted via different routes of infection – 1 is a gram-negative bacillus transmitted via the faecal–oral route, and the other is a protozoan transmitted via the bite of an insect vector – typhoid and malaria share similar clinical and epidemiologic features.

Typhoid fever control efforts have included the establishment of the Typhoid Fever Surveillance in Africa Program (TSAP) in 2009 – a network of 13 sentinel sites in 10 sub-Saharan African countries – created to generate high-quality, contemporary, and standardised data on the incidences of typhoid fever and invasive nontyphoidal salmonellosis in sub-Saharan Africa.^7^ The overarching aim was to produce the evidence required to support policy makers in introducing prevention efforts against invasive *S. typhi* infections – in particular, the introduction of safe and effective typhoid fever vaccines into routine immunisation programmes. TSAP has achieved this ambitious target, finding high incidences of typhoid fever in both rural and urban populations in several countries in sub-Saharan Africa. The results of TSAP will dictate the direction of future typhoid fever research and control endeavours in Africa, and at last provide a key piece of the disease burden jigsaw puzzle.^7^

Malaria control in Tanzania has been significantly scaled up in the past decade. The Tanzanian National Voucher Scheme (TNVS) has expanded the availability and accessibility of insecticide-treated nets (ITNs), particularly for children and pregnant women, by subsidising costs of bed nets for about a decade now.^8^ Parallel to this are other similar programmes aimed at strategically controlling malaria, such as the National Insecticide Treated Nets (NATNETS) Tanzania “Under-five Catch-up Campaign” and “Universal Coverage Campaign”, which have contributed to an effective integrated malaria control environment through increased ITN use, subsidised tests, artemisinin-based medicines, and massive community sensitisation initiatives.^9^ The net result of these strategies has been a reduction in malaria incidence and child mortality rates in Tanzania.^8^

In malaria-endemic areas, the clinical presentation of patients in the early stages of typhoid fever is challenging because it is similar to numerous other causes of febrile illness, such as malaria. Of all symptoms, persistent fever is a prominent source of difficulty when narrowing down the differential diagnosis list.^10^ In most resource-limited tropical settings, definitive laboratory diagnosis of concurrent malaria and typhoid fever is based on blood smear microscopy for malaria and the Widal test or bacterial culture for salmonellosis.

Although the introduction of malaria rapid diagnostic testing (mRDT) has reduced the diagnostic dilemma of patients presenting with fever, challenges exist that limit the use of mRDT, including the logistical challenges involved in increasing coverage to areas not accessible by road. Nonetheless, many endemic countries in sub-Saharan Africa have adopted mRDT to expand parasitological-based malaria diagnosis capacity.^10,11^ The WHO recommends that malaria treatment be based on diagnostic test results, but this directive has yet to gain universal acceptance. This recommendation will become particularly important as the incidence of malaria decreases, as it will become more important to distinguish cases of malaria from other febrile illnesses.^12^

Despite efforts invested in the diagnosis of malaria and typhoid fever coinfections, it is concerning that diagnostic challenges continue to hinder effective malaria and typhoid control in the tropics. This is due to a combination of factors, including the nonspecific clinical presentations of the diseases, the high prevalence of asymptomatic infections in many areas, the lack of resources and insufficient access to trained health-care providers and facilities, and the widespread practice of self-treatment for clinically suspected malaria or typhoid fever.^13–15^ Unfortunately, there is a paucity of epidemiologic data regarding the extent of coinfection with *P. falciparum* and *S. typhi* in many parts of Africa, including Tanzania. Such data could assist clinicians to make more informed decisions when patients present with symptoms that pose a diagnostic dilemma.

We designed this study to provide baseline epidemiologic data on the prevalence of *P. falciparum–S. typhi* coinfections in 2 areas of Northern Tanzania for which *S. typhi* prevalence has not previously been determined. These data will provide useful baseline information that will clarify the magnitude of *P. falciparum–S. typhi* coinfection and facilitate reasonable diagnostic and prescribing decisions.

## METHODS

### Study Design

This community-based, cross-sectional study, which aimed to determine the prevalences of *P. falciparum* and *S. typhi* infection and coinfection and their associations with fever, was conducted from February to June 2016, in Bondo Ward, Tanga Region, and Magugu Ward, Manyara Region, in Northern Tanzania.

### Study Sites

The 2 sites are located about 600 km apart and were selected based on their differing locations, climatic conditions, malaria transmission intensities, and the absence of data on prevalence of *S. typhi* infections in these areas. Magugu is located at 4°12’ S and 35°45’ E and is about 1,392 m above sea level. Bondo is about 309 m above sea level at 5°22’60” N and 38°34’60” E. The natives of both areas are agropastoralists with moderate human–animal interaction. Both areas have 2 rainy seasons per year, with a long rainy season between February and May and short rainy season between October and December. The long rainy seasons are usually followed by high numbers of reported fever cases.^11,16,17^

### Study Population

Study participants consisted of children and adults, aged 1 year and above, who were residents of the study sites, Bondo and Magugu, for at least 6 months and consented to participate in the study.

### Sample Size

The minimum sample size for prevalence determination was estimated using the Epi Tools online sample size calculator and the following formula: [Z^2^p(1-p)]/c^2^, where Z=1.96 for the 95% confidence level, p=the expected true proportion of (9.0%) and c=the minimal tolerable error at the 95% confidence level (0.05). This formula yielded a minimum sample size of 126. We enrolled 128 participants. Following community sensitisation activities, community members were invited to participate, and those who met the enrolment criteria were consecutively recruited into the study until the desired sample size was attained.

### Data Collection and Diagnostic Procedures

A short questionnaire was used to obtain demographic information. Fever was defined as an axillary body temperature ≥37.5°C. Thick and thin blood smears for malaria microscopy were prepared as described elsewhere.^18,19^ Briefly, blood samples of 0.5 to 1 ml were collected into sterile tubes by venepuncture from all consenting participants, and about 10 ml of whole blood was used for mRDT (SD BIOLINE® Malaria Ag P. f/Pan, Suwon City, South Korea) and microscopy. These procedures were carried out by a trained laboratory technician. For each consenting participant, 1 ml of whole blood was aseptically collected into ethylenediaminetetraacetic acid (EDTA) vacutainer tubes. The tubes were shipped to the local laboratory in dry ice and later sent to the Kilimanjaro Christian Research Institute's biotechnology laboratory and stored at –20°C for laboratory analyses. For the purposes of this study, malaria parasitaemia (*P. falciparum* infection) was defined by detection of *P. falciparum* using a real-time polymerase chain reaction (RT-PCR) assay, regardless of the presence of fever or other clinical symptoms. mRDT and microscopy were done to aid clinical decision making and management.

Children under 5 years of age who were found to be malaria-positive by mRDT were treated with antimalarials according to national and WHO guidelines. Adults with fever and positive mRDT results were treated with artemetherlumefantrine, the first-line antimalarial drug in Tanzania. Severe paediatric cases were referred to the nearby district hospital in Korogwe District. Participants who were found to have salmonellosis were appropriately managed with ciprofloxacin according to national guidelines.

### DNA Extraction

DNA extraction and purification were done using QIAamp DNA Mini Kits (Qiagen, Valencia, CA, USA) according to the manufacturer's instructions. A 200 μl aliquot of blood was lysed by QIAGEN protease and bound to a QIAamp membrane by centrifugation according to the manufacturer's instructions. The wash buffers, AW1 and AW2, were sequentially used to remove residual contaminants to improve the purity of the DNA. The purified DNA was then eluted from the QIAamp membrane using buffer AE (Qiagen, Valencia, CA, USA) and stored at –20°C ready for the RT-PCR assay.

### Detection of *P. falciparum* and *S. typhi* by RT-PCR

The RT-PCR assay was carried out in an Applied Biosystems ViiA^TM^ 7 Real-Time PCR system (Thermo Fisher Scientific, Waltham, MA, USA). The Master Mix kit for Tropical Fever Core (Fast Track Diagnostics, Luxembourg) was used to prepare the reaction mix. For a single reaction, 12.5 μl of FTD buffer, 1.5 μl of tropical fever primers and probe mix, and 1μl of enzyme mix were placed into single MicroAmp Optical 8-Tube Strips (Thermo Fisher Scientific, Waltham, MA, USA), compatible with the ViiA^TM^ 7 Real-Time PCR system, followed by 10 μl of sample. The same was done for all samples, the positive control, and the extracted negative control. The detection of pathogens was done at wavelengths of 520 nm for *P. falciparum* Tropical Fever 1 Primers and Probes and 620 for *S. typhi* Tropical Fever 2 Primers and Probes in the Master Mix of the Tropical Fever Core kit (Fast Track Diagnostics, Luxembourg). The positive, negative, and internal controls used in this assay were commercially prepared by Fast Track Diagnostics. The positive control contained plasmids for the detection of *P. falciparum* and *S. typhi*. The negative control contained lysis buffer, and the internal control contained *Streptococcus equi*, which was also used as an extraction control. At the end of the run, amplification plots were reviewed in order to adjust the threshold line above all the background noise, as per the manufacturer's instructions.

### Data Processing and Statistical Analysis

Data were analysed using Stata, version 14 (StataCorp, College Station, TX, USA) and categorised into demographic (age, sex, residence) and clinical (body temperature and prevalence of *P. falciparum* and *S. typhi* infections) variables. The participant age groups for analysis were: <5 years, 5 to 9 years, 10 to 15 years, and above 15 years. Descriptive statistics were used to summarise the demographic and clinical characteristics of study participants. The chi-square (X^2^) test was used to determine associations between categorical data. Fisher's exact test was used in cases when expected counts were less than 5. A 2-tailed P value of .05 or less was considered statistically significant.

### Ethical Considerations

Ethical approval was obtained from the Kilimanjaro Christian Medical University College Research Ethical Review Committee (certificate of clearance #2030). Permission was also obtained from community and local authorities. Written informed consent was obtained from each participant before enrolment, and legal guardians consented for minors. Access to the data – which were anonymised – was restricted to the research team.

## RESULTS

### Demographic and Clinical Characteristics

Demographic data are presented in **Table 1**. A total of 128 participants were enrolled, with almost equal enrolment from each site: 63 (49.2%) participants from Bondo and 65 (50.8%) from Magugu. About two-thirds (n=85, 66.4%) were females, and a similar proportion (n=83, 64.8%) were above 15 years old. There were 21 (16.4%) children younger than 5 years of age. Few participants (n=10, 8.1%) were found to have fever at the time of the survey.

**TABLE 1.** Demographic and Clinical Characteristics (N=128)

Variable	n (%)
**Sex**	
Male	43 (33.6)
Female	85 (66.4)
**Site**	
Bondo	63 (49.2)
Magugu	65 (50.8)
**Age, years**	
<5	21 (16.4)
5–9	17 (13.3)
10–15	3 (5.5)
>15	83 (64.8)
**Fever status**^a^	
No	114 (91.9)
Yes	10 (8.1)

a There were 4 missing entries for fever status (n=124).

### Prevalence of *P. falciparum* and *S. typhi* Infection and Coinfection

**Table 2** shows both the overall and site-specific prevalences of *P. falciparum* and *S. typhi* infection. Sixty-three participants were screened for *P. falciparum* infection in Bondo, of whom 31 (49.2%) had malaria parasitaemia, in contrast with the absence of *P. falciparum* parasitaemia in Magugu. The prevalences of *S. typhi* infection in Bondo and Magugu were 17.5% and 9.2%, respectively. The prevalence of *P. falciparum–S. typhi* coinfection was 9.5% in Bondo, and – with no cases of coin-fection in Magugu – the overall coninfection prevalence was 4.7%.

**TABLE 2.** Prevalences of *Plasmodium falciparum* infection, *Salmonella typhi* infection, and *P. falciparum–S. typhi* Coinfection

	Prevalence		
Variable	Bondo (n=63) n (%)	Magugu (n=65) n (%)	Overall (N=128) n (%)
***P. falciparum–S. typhi* coinfection**			
Negative	57 (90.5)	65 (100)	122 (95.3)
Positive	6 (9.5)	0 (0.0)	6 (4.7)
***S. typhi* infection**			
Negative	52 (82.5)	59 (90.8)	111 (86.7)
Positive	11 (17.5)	6 (9.2)	17 (13.3)
***P. falciparum* infection**			
Negative	32 (50.8)	65 (100)	97 (75.8)
Positive	31 (49.2)	0 (0.0)	31 (24.2)

### Association of *P. falciparum* and *S. typhi* Infection With Demographic Characteristics

**Table 3** shows findings related to the associations between *P. falciparum* and *S. typhi* infection and participant demographic variables. When data from both sites were combined, only age was significantly associated with *S. typhi* infection (X^2^=2.1; *P*=.045), with 17.6% and 28.6% of the participants in the 5-to 9-year and 10-to 15-year age groups testing positive for *S. typhi* infection, respectively. Children younger than 5 years had the lowest prevalence of *S. typhi* infection. There was no association between *S. typhi* infection with either participant sex or study site.

**TABLE 3.** Association Between *Plasmodium falciparum* and *Salmonella typhi* Infection With Age, Sex, and Study Site

		*S. typhi* Test Result			
Infection Type	Variable	Negative n (%)	Positive n (%)	Total n	X^2^ (*P* Value)
*S. typhi* infection	**All subjects**	111 (86.7)	17 (13.3)	N=128	
	**Age, years**				
	<5	19 (90.5)	2 (9.5)	21	2.1 (.045)
	5–9	14 (71.4)	3 (17.6)	17	
	10–15	5 (71.4)	2 (28.6)	7	
	>15	73 (88.0)	10 (12.0)	83	
	**Sex**				
	Female	73 (85.9)	12 (14.1)	85	0.2 (.70)
	Male	38 (88.4)	5 (11.6)	43	
	**Site**				
	Bondo	52 (82.5)	11 (17.5)	63	1.9 (.17)
	Magugu	59 (90.8)	6 (9.2)	65	

		*P. falciparum* Test Result			
		Negative n (%)	Positive n (%)		
*P. falciparum* infection	**All subjects**	97 (75.8)	31 (24.2)	128	
	**Age, years**^a^				
	<5	14 (66.7)	7 (33.3)	21	18.2 (<.001)
	5–9	8 (47.1)	9 (57.9)	17	
	10–14	3 (42.9)	4 (57.1)	7	
	>15	72 (86.8)	11 (13.3)	83	
	**Sex**				
	Female	64 (75.3)	21 (24.7)	85	0.03 (.86)
	Male	33 (76.7)	10 (23.3)	43	
	**Site**				
	Bondo	32 (50.8)	31 (49.2)	63	b
	Magugu	65 (100)	0 (0.0)	65	

a Fisher's exact test performed.

b Statistical analysis not performed because 1 of the sites had a prevalence of 0 (0.0%).

Similarly, there was a strong association between *Plasmodium* infection and age (X^2^=18.2; *P*<.001), with the 5-to 9-year and 10-to 15-year age groups having parasitaemia prevalences of 57.9% and 57.1%, respectively. The participants aged >15 years had the lowest prevalence of *P. falciparum* infection. We did not find any association between *P. falciparum* infection and sex.

### Factors Associated With *P. falciparum–S. typhi* Coinfection and Fever

**Table 4** shows that the associations between *P. falciparum–S. typhi* coinfection and sex, age, and study site were all not statistically significant.

**TABLE 4.** Factors Associated With *Plasmodium falciparum* and *Salmonella typhi* Coinfection

	*Plasmodium–Salmonella* Coinfection			
Variable	Negative n (%)	Positive n (%)	Total n	X^2^ (*P* Value)
**All subjects**	122 (95.3)	6 (4.7)	N=128	
**Age, years**^a^				
<5	20 (95.2)	1 (4.8)	21	4.3 (.10)
5–9	15 (88.2)	2 (11.8)	17.0	
10–14	6 (85.7)	1 (14.3)	7	
>15	81 (97.6)	2 (2.4)	83	
**Sex**^a^				
Female	81 (95.3)	4 (4.7)	85	0.0 (1.0)
Male	41 (95.4)	2 (4.6)	43	
**Site**				
Bondo	57 (90.5)	6 (9.5)	63	b
Magugu	65 (100)	0 (0.0)	65	

a Fisher's exact test performed.

b Association test not performed because 1 of the sites had no cases of coinfection.

**Table 5** shows the analysis findings related to the associations between various factors and fever. Seven (23.3%) of 30 participants with *P. falciparum* parasitaemia had fever, compared with only 2 (12.5%) who had fever among the 16 participants who tested positive for *S. typhi* infection. *P. falciparum* infection was associated with fever (X^2^=12.4; *P*<.001), and no association was found between *S. typhi* infection and fever. Among the 6 participants with *P. falciparum–S. typhi* coinfection, 2 (33.3%) had fever (X^2^=5.5; *P*=.019). Fever was most prevalent among 5-to 15-year-olds (X^2^=17.44, *P*<.001). There was no association between sex and fever.

**TABLE 5.** Association of Fever With Demographic Characteristics and Infection With Plasmodium falciparum, Salmonella typhi, or Both (N=124)

	Fever Status^a^		
Variable	Fever^b^ n(%)	No Fever n (%)	X^2^ (*P* Value)
***P. falciparum* infection**			
Positive	7 (23.3)	23 (76.7)	12.4 (<.001)
Negative	3 (3.2)	91 (96.8)	
***S. typhi* infection**			
Positive	2 (12.5)	14 (87.5)	0.5 (.48)
Negative	8 (7.3)	101 (92.7)	
***P. falciparum–S. typhi* Coinfection**			
Positive	2 (33.3)	4 (66.7)	5.5 (.019)
Negative	8 (6.7)	111 (93.3)	
**Residence**			
Bondo	10 (16.1)	52 (83.9)	^c^
Magugu	0 (0.0)	62 (100.0)	
**Age**^d^			
<5	3 (15.8)	16 (84.2)	17.4 (<.001)
5–9	5 (29.4)	12 (70.6)	
10–15	1 (14.3)	6 (85.7)	
>15	1 (1.2)	80 (98.8)	
**Gender**			
Male	5 (12.8)	34 (87.2)	1.7 (.19)
Female	5 (5.9)	80 (94.1)	

a There were 4 missing entries for fever status (n=124).

b Fever was defined by axillary temperatures ≥37.5°C.

c Association test not performed because 1 of the sites had no cases of coinfection.

d Fisher's exact test performed.

## DISCUSSION

The high prevalence of pathogens that cause overlapping clinical signs and symptoms, particularly fever, poses a serious challenge in diagnosing and managing febrile illness. This is particularly true in resource-poor countries, such as Tanzania, where the diagnostic infrastructure is constrained. Diagnoses are frequently provisional in the absence of adequate confirmatory tests, and it is, therefore, a common practice that antimicrobials are irrationally prescribed, leading to serious consequences, including the development of antimicrobial resistance.^20,21^ This scenario justifies the application of high-throughput and highly sensitive molecular tools to determine the causes of diseases with overlapping clinical signs and for proper management of febrile illness.

About half of the participants in Bondo had *P. falciparum* parasitaemia. This finding represents an increase in malaria parasitaemia prevalence compared with what has been observed in the past decade, during which time significant shifts have been reported. In 2009, the *P. falciparum* parasitaemia prevalence was 32.8% in the rainy season^22^; the prevalence dropped to 12% in 2011,^12^ with a further drop to 8.6% in 2013.^23^ However, a recent survey conducted in Bondo in 2016 reported a prevalence of 20.5%.^18^ In the absence of studies to explain the observed fluctuations in malaria prevalence in the study area, a number of explanations can be proposed regarding the outcomes of malaria control efforts implemented in the studied areas, including seasonal differences at the times when data were collected for the respective studies.

In the past decade, the TNVS, implemented by the Tanzanian government, has contributed to a significant reduction in malaria through increasing the availability and accessibility of ITNs mainly targeting children and pregnant women.^8^ Other initiatives, such as the NATNETS Tanzania “Under-five Catch-up Campaign” and “Universal Coverage Campaign”, have complemented the TNVS.^9^ These initiatives have contributed to a substantial reduction in the incidence and prevalence of malaria in Tanzania, as reported by recent studies.^8,9^ However, a substantial impact on the disease burden has not been sustained. Challenging obstacles preventing optimal malaria control persist, including poor access to health care, poor performance of health service delivery, poor availability of proper diagnosis and treatment services, increased drug resistance, and high costs of health-care services.^15^ The increased malaria prevalence in Bondo suggests the possibility of a breakdown of malaria control strategies in the area. Support for malaria control at both the national and district levels has increased considerably over the past few years. The low prevalence of *P. falciparum* infection in Magugu has been reported for about a decade,^11,20,24^ likely because Magugu serves as an experimental study site for a national pesticide research institute.^25^

The prevalence of *S. typhi* infection in Bondo was high, with about one-fifth of the population testing positive for *S. typhi*, while in Magugu, about one-tenth of individuals were found to be infected with *S. typhi*. Previous studies have reported a similar uneven distribution of *Salmonella* infection, with the occurrence of invasive nontyphoidal salmonellosis more common in areas with high malaria transmission rates, while *S. typhi* is reportedly common in areas with low rates of malaria transmission.^26^ Wide variations in the prevalence of *S. typhi* infection have been reported in different parts of Tanzania and elsewhere in sub-Saharan Africa.^19,27,28^

Participants between 5 and 15 years of age had the highest *S. typhi* infection prevalence in this study – an observation that was statistically significant. *S. typhi* infection was not associated with sex or study site. It has been reported that the most common dietary protein sources in Tanzania include milk, eggs, and meat, with urban dwellers consuming more of these products than rural inhabitants.^29^ These types of food are known important risk factors for human salmonellosis worldwide.^30^ Although the consumption of these foods is generally low in Tanzania, efforts have been made to ensure that school-aged children consume milk countrywide.^31^ Whether sanitary precautions are strictly observed or not, this could serve as a possible source of *S. typhi* infection in this age group, especially in periurban sites like Bondo. We found that malaria parasitaemia was most prevalent among participants between 5 and 15 years of age. A shift in burden of infection from being more prevalent among children under 5 to being more prevalent among school-aged children has recently been reported.^18^ In the absence of objective data to explain this shift, we speculate that because most malaria interventions over the past decade have targeted children under 5 and pregnant mothers,^8,9^ our findings reflect a reduction of immunity to malaria among older children, who were previously targeted by rigorous malaria control interventions when they were below 5 years of age. For both *S. typhi* and *P. falciparum*, individuals older than 15 years had the lowest infection rates, most likely explained by the build-up of specific immunity to these infections.

The prevalence of *P. falciparum–S. typhi* coinfection was low (4.7%), and coinfection occurred only in Bondo, where the malaria parasitaemia prevalence was high. Despite the low coinfection rate, there are important implications for the coinfected individuals. There is accumulating epidemiologic and preclinical evidence supporting a causal association between malaria and nontyphoidal salmonellosis.^32^ However, the clinical characteristics and consequences of *P. falciparum–S. typhi* coinfection are not well documented, although mortality associated with coinfection has been reported to be higher than that associated with malaria alone.^32^

We found that nearly a quarter of individuals with *P. falciparum* infection in Bondo had fever, implying a significant contribution of *P. falciparum* infection to the development of fever. Besides that, 12.5% of participants with *S. typhi* infection had fever, reflecting an important contribution of *S. typhi* to the burden of febrile illness in the area. One-third of individuals coinfected with *P. falciparum* and *S. typhi* had fever.

We presume that the remaining three-quarters of afebrile participants with *P. falciparum* infection were asymptomatic carriers. However, our findings do not rule out infection with other causes of fever that we did not test for. This possibility is supported by the findings of a previous study conducted in Magugu, where more than two-fifths of patients were clinically misdiagnosed as having malaria even though only less than 1% of blood films were confirmed *P. falciparum*-positive.^11^ Testing for a wider range of fever-causing pathogens would yield more specific findings to inform diagnostic and management guidelines for febrile illnesses. Our findings underscore the urgency of developing appropriate guidelines for the diagnosis and treatment of *P. falciparum–S. typhi* coinfection, as clinicians commonly dismiss the possibility of multiple infections during an initial patient visit. In attempts to implement an intensive malaria control programme in Tanzania, the Ministry of Health has launched a widely advertised Kiswahili slogan: “*Siyo kila homa ni malaria*”, literally translated as, “Not every fever is malaria”. This slogan not only reminds patients to avoid self-medication, it also reminds clinicians to carefully consider mixed infections when patients present with generic symptoms, such as fever and headache.

## RECOMMENDATIONS AND CONCLUSION

We report a prevalence of *P. falciparum–S. typhi* coinfection of 9.5% in Bondo, and we detected no coinfection in Magugu. One-third of the coinfected individuals in Bondo had fever. *P. falciparum* infection was an important contributor to the febrile illness burden in Bondo. Magugu was free from *P. falciparum* infection during the study period. Considering the presence of coinfections with *P. falciparum* and *S. typhi*, we recommend emphasising and enforcing the use of mRDT to reduce irrational use of medications. We also recommend the scale-up of typhoid fever diagnostic tools to underserved areas of Tanzania to help distinguish between malaria and typhoid fever.
